# Androgen receptor expression in a Sri Lankan patient cohort with early breast carcinoma

**DOI:** 10.1186/s12905-020-01068-5

**Published:** 2020-09-14

**Authors:** Harshima Disvini Wijesinghe, Gayani Kokila Wijesinghe, Zahara Mansoor, Sanjeev Vigneshwara, Janakie Fernando, Dehan Gunasekera, Menaka Dilani Samarawickrama Lokuhetty

**Affiliations:** 1 Department of Pathology, Faculty of Medicine, University of Colombo, Colombo, Sri Lanka; 2 Department of Pathology, National of Hospital of Sri Lanka, Colombo, Sri Lanka; 3 Apeksha Hospital, Maharagama, Sri Lanka

**Keywords:** Breast carcinoma, Androgen receptors, Prevalence, Prognosis, Clinicopathological features, Sri Lanka

## Abstract

**Background:**

Androgen receptor (AR) expression is emerging as a prognostic biomarker in breast carcinoma (BCa). The study aimed to determine the prevalence of AR expression by immunohistochemical analysis among a cohort of Sri Lankan women with early BCa and to evaluate its association with clinicopathological features including immunohistochemical molecular subtype and early survival.

**Method:**

We studied the clinical and pathological features and immunohistochemical profile of 141 women undergoing primary surgery for early BCa, followed by standard adjuvant therapy. AR status was assessed by immunohistochemistry in all cases. Overall survival (OS) and disease-free survival (DFS) was determined. The relationship between AR expression and clinical and pathological parameters and immunohistochemical molecular subtype was analyzed using Student T test and chi-square tests. Cox regression analysis was used to analyze the prognostic impact of AR expression.

**Results:**

AR expression was seen in 40.8%(95%CI 33.10–49.07%) of the BCa study cohort. None of the clinical data studied showed a significant association with the AR status(*p* > 0.05). Ductal carcinoma in situ(*p* = 0.003), oestrogen receptor (ER) (*p* = 0.001) and progesterone receptor (PR) (*p* = 0.001) positivity and luminal IHC molecular subtype(*p* = 0.016) were significantly associated with AR-positive status. AR-negative status was significantly associated with tumour necrosis > 50%(*p* = 0.031), moderate to extensive lymphocytic infiltrate at the tumour margin(*p* = 0.025) and basal triple negative breast carcinoma(*p* = 0.016).

The mean duration of patient follow-up was 46.70(95% CI 46.495–46.905) months (3.89 years). On univariate analysis, AR-positivity was associated with better OS among ER-positive tumours(*p* = 0.047), specifically in postmenopausal women (*p* = 0.030). In ER-negative tumours, AR positivity was associated with worse DFS (*p* = 0.036). On multivariate analysis, TNM stage and ER/AR status were predictive of survival. ER-positive/AR-positive (ER+/AR+) tumours demonstrated better OS than ER-positive/AR-negative (ER+/AR-) tumours(*p* = 0.015). ER-negative/AR-positive (ER−/AR+) tumours (*p* = 0.014) had a worse DFS than ER-negative/AR-negative (ER−/AR-) tumours.

**Conclusions:**

AR prevalence obtained was low. AR positivity was associated with positivity for ER and PR. On multivariate analysis, apart from TNM stage only ER/AR status were predictive of OS and DFS, with concordant expression of ER/AR demonstrating a better, early survival.

## Background

Breast carcinoma (BCa) is the commonest malignancy among women in Sri Lanka and worldwide. In 2010, 27% of women diagnosed with cancer in Sri Lanka had BCa with a lifetime risk of 2.5% in the population [1].

BCa can be categorized into several molecular subtypes based on the surrogate immunohistochemical marker expression. Oestrogen receptors (ER), progesterone receptors (PR), HER2, Ki67 and basal markers (CK5/6, CK14, CK17, 34βE12 and EGFR) are analyzed by immunohistochemistry (IHC). Based on these results the tumours are categorized into Luminal A, Luminal B, HER2 enriched, triple negative and basal like subtypes [2, 3]. The IHC defined molecular subtype of a tumour has a significant impact on treatment decisions. It is predictive of response to treatment and important in the assessment of prognosis [4].

Expression of androgen receptors (AR) in BCa has generated considerable interest in the recent past as a prognostic biomarker. The human androgen receptor *AR* gene is located in the X chromosome at position Xq11–12. It is known to produce its effects through 3 major pathways which contribute to AR function in BCa: genomic signaling, non-genomic signaling and signaling via cross talk with growth factors and cytokines [5]. Different signaling pathways are seen in the different molecular subtypes of BCa. In ER-positive BCa, AR signaling often antagonizes the growth stimulatory effect of ER signaling; in luminal AR subtype of TNBC identified by molecular assays, AR seems to drive tumor progression; in HER2-positive BCa, in the absence of ER expression, AR signaling has a proliferative role [6]. This explains why the prognostic impact of AR has been shown to be dependent on the molecular subtype of tumour [7–10]. It is also the rationale behind the use of androgen agonists in some AR-positive/ER-positive tumors (AR+/ER+) and AR antagonists in triple-negative/AR-positive tumors (TNBC/AR+) and a combination of AR antagonists and anti-HER2 agents or other signaling pathway inhibitors in HER2-positive/AR- positive (HER2+/AR+) tumors [6, 11].

Systematic reviews and meta-analysis have shown AR positivity to be associated with favorable clinical outcomes [12, 13]. However these studies did not provide evidence on prognostic relevance of AR in different breast cancer subtypes. A correct understanding of the prognostic value of AR in each breast cancer subtype would be of value in refining the prognostic and predictive outcomes in each specific subgroup of early breast cancer patients. It is also crucial for the development of AR agonist and antagonist therapies.

Data regarding the prevalence and the impact of AR expression in BCa and survival are mostly from the west and none from Sri Lanka. Only a few studies have been done in Asia; in Iran, India, Thailand and China [14–17].

We aimed to determine the prevalence of androgen receptor expression by immunohistochemical analysis among a cohort of Sri Lankan women with early breast carcinoma, and to evaluate its association with clinical and pathological features including immunohistochemical molecular subtype and early survival.

## Methods

### Sample selection

Ethical approval for the study was obtained from the Ethical Review Committee of the Faculty of Medicine, University of Colombo (EC-17-088). The study population comprised all women undergoing surgery for invasive breast carcinoma at the National Hospital of Sri Lanka from June 2012 to December 2014. Inclusion criteria were women undergoing mastectomy or wide-local excision for an invasive breast carcinoma of early stage. Early stage breast carcinoma was defined as cancer confined to the breast with or without regional lymph node involvement, and absent distant metastasis [18]. Patients with mixed in situ and invasive carcinoma were included. Poor tumour fixation and neoadjuvant chemotherapy have been shown to alter histopathological and ER/PR and HER2 receptor status of BCa [19]. Therefore women with BCa undergoing surgery following neoadjuvant therapy and tumours with evidence of poor fixation (suboptimal tumour preservation involving ≥80% of the tumour) were excluded. Other exclusion criteria were lack of follow-up data, non-availability of tissue blocks and cases with microcarcinoma having inadequate invasive carcinoma for analysis of all histopathological parameters.

### Sample size

The study population comprised 301 patients. One hundred and forty one cases were selected from 179 cases fulfilling the inclusion and exclusion criteria.

### Evaluation of clinicopathological parameters

The clinical parameters evaluated included ethnicity, age, body mass index, parity, history of breast feeding, menopausal status, family history of BCa and use of oral contraceptives and hormone therapy. The clinical data was collected by the investigators through patient interviews. The following information was retrieved from the pathology reports: type of surgery, tumour size, margin involvement and lymph node status. Slides of cases that had been reported as having a positive margin were reviewed to confirm the presence of margin involvement. Only cases showing the presence of tumour on the ink were classified as having a positive margin. The haematoxylin and eosin stained slides of the tumour were evaluated by the investigators for the following parameters: histological type, histological grade, necrosis, ductal carcinoma in situ (DCIS), lymphovascular invasion, tumour margins, degree of lymphoid infiltrate at the tumour-host interface and within the tumour, degree of desmoplasia/hyalinization at the tumour-host interface and within the tumour, vascular density at the tumour-host interface, cell margins and calcification. Histological subtyping was done according to the 4th World Health Organization Classification of Tumours of the Breast [20]. The Elston-Ellis modification of Scarff-Bloom-Richardson grading system [21] was used to determine the histological grade. Tumour margins were assessed qualitatively and categorized as pushing or infiltrative. A pushing margin was defined as a broad well defined margin with tumour cells arranged in clusters or islands at the tumour-host interface. The tumour and tumour-host interface were screened on X10 magnification to assess the degree of lymphoid infiltrate and desmoplasia. These were assessed semi-quantitatively. They were categorized as absent to mild (lymphoid infiltrate/desmoplasia ≤1/3 of the area assessed) and moderate to severe (lymphoid infiltrate/desmoplasia involving > 1/3 of the area assessed. The vascular density at the tumour host interface was assessed in the area of highest vascular density and categorized into two categories (< 5 blood vessels/medium power field and ≥ 5 blood vessels/medium power field. A medium power field was a field with a field diameter of 0.2 mm on × 10 magnification.

### Immunohistochemical analysis

Immunohistochemical staining for ER, PR, HER2 and AR were performed by investigators on representative paraffin embedded tissue in all selected cases. CK5/6, EGFR, 34ßE12 and CK14 were evaluated in the cases found to be triple negative (Negative for ER, PR and HER2). Relevant negative and positive control slides were included with each batch of slides stained with immunohistochemical markers.

The antibodies, dilution and the method used and interpretation of IHC stained sections are shown in Table 1. All cases were evaluated by the investigators. The cut-off point used for positivity versus negativity for ER, PR or AR status was greater than or equal to 1% of tumour cells [19, 22]. The ASCO - CAP guideline of 2013 was used for the interpretation of HER2 [23]. Tumours that were equivocal for HER2 (HER2–2+) by IHC were further evaluated by by fluorescent in situ hybridization (FISH) to determine the HER2 status.

**Table 1:** Immunohistochemical protocols and scoring for ER, PR, AR, HER2 and basal markers

Marker	Supplier	Clone	Antigen retrieval	Primary dilution	Amplification step	Interpretation	Binarized as positive	Binarized as negative
ER	Dako	M7047 Clone ID5	Pressure cooker in citrate buffer	1:60	Universal secondary antibody Envision (Dako K5007)	ASCOCAP 2010 guideline [22]	> = 1% of tumour cells positive	< 1% of tumour cells positive
PR	Dako	M3569 Clone PgR636	Pressure cooker in citrate buffer	1:50		ASCOCAP 2010 guideline [22]	> = 1% of tumour cells positive	< 1% of tumour cells positive
HER2	Dako	A0485	Microwave in citrate buffer	1:600		ASCOCAP 2013 guideline [23]	HER2 = 3+	HER2 = 0,1
CK5/6	Dako	M7237 Clone D5/16B4	Pressure cooker in citrate buffer	1:25		Proportion Score 0 = No cytoplasmic staining 1-Positive cytoplasmic staining in 1–33% of cells 2-Positive cytoplasmic staining in 33–66% of cells 3-Positive staining in the cytoplasm in > 66% of cells Intensity Score 0-No cytoplasmic staining 1-Weak cytoplasmic staining 2-Moderate cytoplasmic staining 3Strong cytoplasmic staining	Total score ≥ 2	Total score < 2
34βE12	Dako	N1553 Clone 34βE12	Microwave in Dako Target Retrieval Solution (S1700)	Undiluted				
CK14	Cell Marque	LL002	Pressure cooker in citrate buffer	1:100				
EGFR	Dako	M7239 Clone E30	Proteolytic digestion with Dako Proteinase K (S3020)	1:25		Atkins [24] et al.	Any membrane staining above background, whether or not it is completely circumferential.	No membrane staining above background in any tumor cell
**AR**	**Dako**	**M3562** **Clone AR 441**	**Pressure cooker in citrate buffer at pH 6 for 7–8 min**	**1:50**		**ASCOCAP 2010 guideline on immunohistochemical testing of ER/PR** [22]	**> = 1% of tumour cells positive**	< 1% of tumour cells positive

The tumours were broadly categorized into luminal, HER2 and triple negative breast carcinoma (TNBC) groups based on ER, PR and HER2 status. A proportion of the luminal breast carcinomas could have been further subclassified as luminal B, based on positivity for HER2 [3]. However, further classification of the luminal subgroup into luminal A and B was not attempted as the proliferative index by Ki67 was not evaluated in all cases. The triple negative tumours were subdivided into two groups based on the expression of basal markers, i.e. non-basal triple negative (basal markers negative) and basal triple negative (basal markers positive).

### Collection of survival data

All patients had been treated and managed according to standard protocols [25] with 119 women undergoing mastectomy and 22 undergoing wide local excision respectively. Subsequent adjuvant therapy options included combinations of hormonal therapy with anti oestrogenic agents (in ER and/or PR positive tumours), antiHER2 therapy (in HER2 positive tumours), chemotherapy and radiotherapy. Androgen receptor agonists and antagonists were not used in the treatment. Treatment received, early overall survival (OS) and disease-free survival (DFS) at short term was determined from patient interviews and review of clinic records.

### Statistical analysis

Proportions and 95% confidence intervals were calculated to estimate prevalence. The relationship between clinicopathological parameters, IHC molecular subtype and the AR expression was analyzed using Student T test and chi-square tests. A *p* value of < 0.05 was taken as significant. Univariate Cox regression analysis was used to analyze the prognostic impact (OS and DFS) of AR expression on the entire sample as a whole and also when stratified according to histological grade, ER status and menopausal status. Multivariate Cox regression analysis was performed to analyse factors affecting OS and DFS. Statistical Package for the Social Sciences SPSS (IBM Corp. Released 2016. IBM SPSS Statistics for Windows, Version 20.0. Armonk, NY: IBM Corp.) was used for data analysis.

## Results

### Characteristics of the study sample

The characteristics of the study sample are summarized in Table 2. The mean age of the cohort was 55.46 years (95% CI 53.6–57.3 years) ranging from 29 to 77 years. The majority were postmenopausal (97/148–68.8%) and overweight (Mean BMI 26.69 kg/m^2^–95% CI 25.7–27.7 kg/m^2^). Eleven (7.8%) had a family history of breast carcinoma.

**Table 2:** Clinical and pathological characteristics of the study sample

Clinico-pathological feature		Mean (SD) or Number (Percentage)
Age	Mean age (years)	55.46 (11.28)
Body Mass Index	Mean BMI (kg/m^2^)	26.69 (6.24)
Ethnicity	Sinhalese	115 (81.6%)
	Tamil	13 (9.2%)
	Moor	13 (9.2%)
Menopausal status	Premenopausal	44 (31.2%)
	Post-menopausal	97 (68.8%)
Parity	Nulliparous	14 (9.9%)
	Parous	122 (86.5%)
	Not available	5 (3.5%)
History of oral contraceptive use	Present	27 (19.1%)
	Absent	109 (77.3%)
	Not available	5 (3.5%)
History of use of hormone replacement therapy	Present	2 (1.4%)
	Absent	133 (94.3%)
	Not available	6 (4.3%)
History of breast feeding	Present	120 (85.1%)
	Absent	16 (11.3%)
	Not available	5 (3.5%)
Family history of breast cancer	Present	11 (7.8%)
	Absent	130 (92.2%)
TNM stage*	Stage I	17 (12.1%)
	Stage II	93 (66.0%)
	Stage III	31 (22.0%)
Nodal stage* *(Lymph nodes were assessed in 128 patients)*	N0	72 (56.2%)
	N1	26 (20.3%)
	N2	14 (10.9%)
	N3	16 (12.5%)
Histology type	Ductal	125 (88.7%)
	Lobular	6 (4.3%)
	Mucinous	3 (2.1%)
	Micropapillary	1 (0.7%)
	Metaplastic	4 (2.8%)
	Adenoid cystic	1 (0.7%)
	Carcinoma with neuroendocrine features	1 (0.7%)
Histology grade	Grade 1	31 (22.0%)
	Grade 2	51 (36.2%)
	Grade 3	59 (41.8%)
Receptor status		
ER	Positive	68 (49.3%)
	Negative	70 (50.7%)
PR	Positive	75 (53.6%)
	Negative	65 (46.4%)
HER 2	Positive	20 (14.9%)
	Negative	121 (85.1%)
Molecular subtypes	Luminal	87 (61.7%)
	HER 2	17 (12.1%)
	Triple negative	37 (26.2%)
Type of surgery	Wide – local excision	22 (15.6%)
	Mastectomy	119 (84.4%)
Type of adjuvant therapy *(Specific treatment details were available in 84 patients)*	Hormonal treatment	50 (59.5%)
	Chemotherapy	32 (38.1%)
	Trastuzumab	10 (11.9%)
	Radiotherapy	9 (10.7%)

### Prevalence of AR expression

**Fig. 1:**
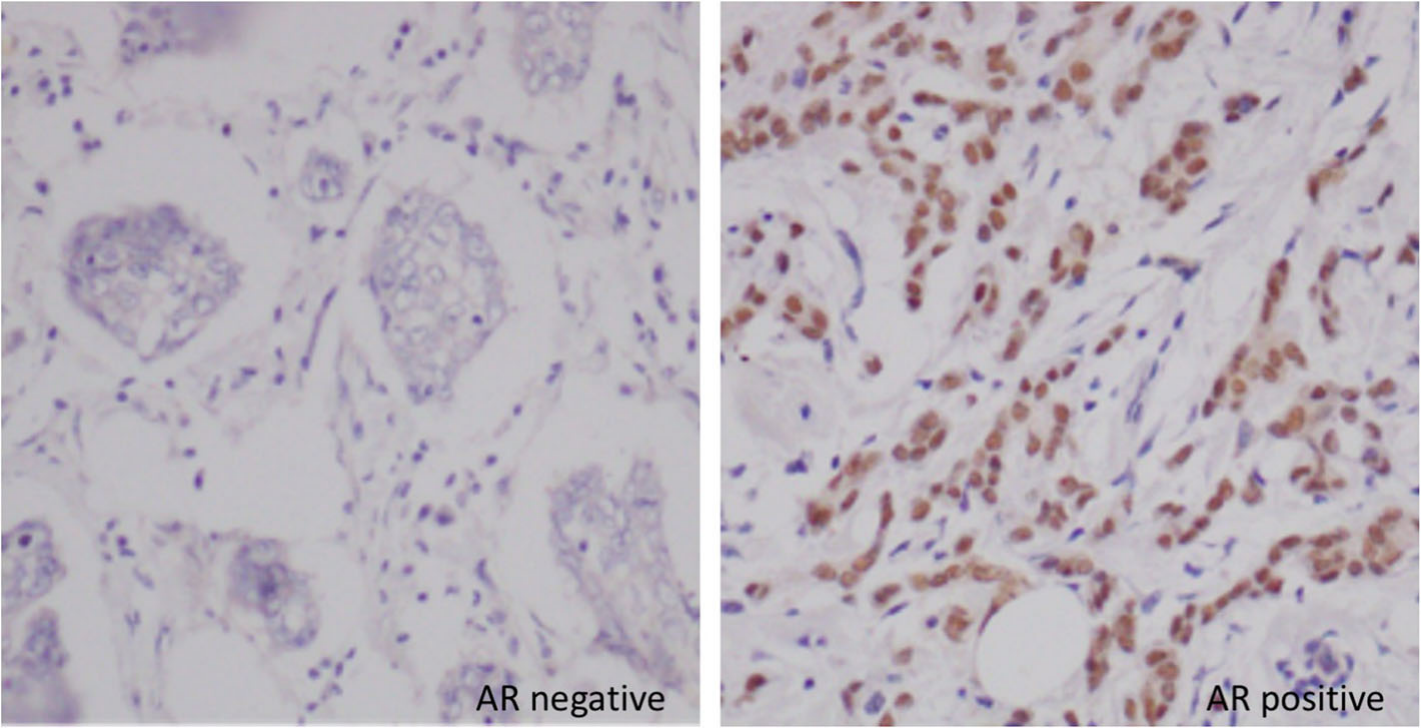
Immunohistochemical staining for AR in breast carcinoma

Positive AR expression (Fig. 1) was seen in 40.8% (95% CI 33.10–49.07%) of the study cohort.

### Association of AR expression with clinical and pathological features

None of the clinical data studied showed a significant association with either AR-positive or negative status (*p* > 0.05) (Table 3). Of the pathological features studied DCIS (*p* = 0.003) and positivity for ER (*p* = 0.001) and PR (*p* = 0.001) were associated with AR positivity. AR-negativity was significantly associated with necrosis > 50% (*p* = 0.031) and a moderate to extensive lymphocytic infiltrate at the tumour margin (*p* = 0.025) (Table 4).

**Table 3:** Association of clinical features and AR status

Clinical feature		AR Status		***p***-value (Student t – test, chi square)
		Positive	Negative	
Age (years)	Mean age (years)	57.19	54.20	0.125^a^
Body Mass Index	Mean BMI (kg/m^2^)	26.82	26.61	0.863^a^
Ethnicity	Sinhalese	44 (77.2%)	71 (84.5%)	0.503^b^
	Tamil	7 (12.3%)	6 (7.1%)	
	Moor	6 (10.5%)	7 (8.3%)	
Menopausal status	Premenopausal	13 (22.8%)	31 (36.9%)	0.076^b^
	Post-menopausal	44 (77.2%)	53 (63.1%)	
Parity	Nulliparous	8 (14.0%)	6 (7.1%)	0.405^b^
	Parous	47 (82.5%)	75 (89.3%)	
	Not available	2 (3.5%)	3 (3.6%)	
History of oral contraceptive use	Present	9 (15.8%)	18 (21.4%)	0.498^b^
	Absent	45 (78.9%)	64 (76.2%)	
	Not available	3 (5.3%)	2 (2.4%)	
History of use of hormone replacement therapy	Present	2 (3.5%)	0 (0%)	0.195^b^
	Absent	52 (91.2%)	81 (96.4%)	
	Not available	3 (5.3%)	3 (3.6%)	
History of breast feeding	Present	46 (80.7%)	74 (88.1%)	0.390^b^
	Absent	9 (15.8%)	7 (8.3%)	
	Not available	2 (3.5%)	3 (3.6%)	
Family history of breast cancer	Present	3 (5.3%)	8 (9.5%)	0.355^b^
	Absent	54 (94.7%)	76 (90.5%)	

^a^Statistical test - Student T test. ^b^Statistical test – Chi square test

Percentages have been calculated to represent the prevalence of each clinical feature within each category of AR status

**Table 4:** Association of pathological parameters and AR expression

Pathological Parameter		AR expression		***p***-value (Chi - square)
		Positive	Negative	
Histological type	Ductal	49^a^ (86.0%)	76^a^ (90.5%)	0.657
	Lobular	3^a^ (5.3%)	3^a^ (3.6%)	
	Mucinous	2^a^ (3.5%)	1^a^ (1.2%)	
	Micropapillary	0^a^ (0%)	1^a^ (1.2%)	
	Metaplastic	2^a^ (3.5%)	2^a^ (2.4%)	
	Adenoid cystic carcinoma	0^a^ (0%)	1^a^ (1.2%)	
	Carcinoma with neuroendocrine features	1^a^ (1.8%)	0^a^ (0%)	
T stage	T1	11^a^ (19.3%)	13^a^ (15.5%)	0.593
	T2	41^a^ (71.9%)	65^a^ (77.4%)	
	T3	5^a^ (8.8%)	6^a^ (7.1%)	
N stage	N0	27^a^ (55.1%)	45^a^ (57.0%)	0.452
	N1	11^a^ (22.4%)	15^a^ (19.0%)	
	N2	7^a^ (14.3%)	7^a^ (8.9%)	
	N3	4^a^ (8.2%)	12^a^ (15.2%)	
TNM stage	Stage 1	8^a^ (14.0%)	9^a^ (10.7%)	0.797
	Stage 11	36^a^ (63.2%)	57^a^ (67.9%)	
	Stage 111	13^a^ (22.8%)	18^a^ (21.4%)	
Histological Grade	Grade 1	15^a^ (26.3%)	16^a^ (19.0%)	0.497
	Grade 2	21^a^ (36.8%)	30^a^ (35.7%)	
	Grade 3	21^a^ (36.8%)	38^a^ (45.2%)	
**Ductal carcinoma in situ**	**Absent**	**21**^**a**^ **(36.8%)**	**52**^**b**^ **(61.9%)**	0.003*
	**Present**	**36**^**a**^ **(63.2%)**	**32**^**b**^ **(38.1%)**	
**Necrosis**	Absent	38^a^ (66.7%)	48^a^ (57.1%)	0.031*
	Focal	15^a^ (26.3%)	16^a^ (19.0%)	
	**Moderate-extensive**	4^a^ **(7.0%)**	20^b^ **(23.8%)**	
Tumour margin	Pushing	43^a^ (75.4%)	64^a^ (76.2%)	0.918
	Infiltrative	14^a^ (24.6%)	20^a^ (23.8%)	
Lympho vascular invasion	Absent	48^a^ (84.2%)	76^a^ (90.5%)	0.262
	Present	9^a^ (15.8%)	8^a^ (9.5%)	
Central desmoplasia/Hyalinization	Absent to mild	24^a^ (42.1%)	40^a^ (47.6%)	0.519
	Moderate-extensive	33^a^ (57.9%)	44^a^ (52.4%)	
Desmoplasia/hyalinisation at edge	Absent to mild	43^a^ (75.4%)	68^a^ (81.0%)	0.432
	Moderate to extensive	14^a^ (24.6%)	16^a^ (19.0%)	
Lymphoid infiltrate in centre	Absent to mild	49^a^ (86.0%)	66^a^ (78.6%)	0.267
	Moderate to extensive	8^a^ (14.0%)	18^a^ (21.4%)	
**Lymphoid infiltrate at edge**	**Absent to mild**	**46**^**a**^ **(80.7%)**	**53**^**b**^ **(63.1%)**	0.025*
	**Moderate to extensive**	**11**^**a**^ **(19.3%)**	**31**^**b**^ **(36.9%)**	
Vascular density at edge	< 5 vessels/mpf	16^a^ (28.1%)	21^a^ (25.0%)	0.684
	≥5 vessels/mpf	41^a^ (71.9%)	63^a^ (75.0%)	
Calcification	Absent	52^a^ (91.2%)	79^a^ (94.0%)	0.522
	Present	5^a^ (8.8%)	5^a^ (6.0%)	
Cell margins	Distinct	26^a^ (45.6%)	42^a^ (50.0%)	0.609
	Indistinct	31^a^ (54.4%)	42^a^ (50.0%)	
**ER status**	**Positive**	**36**^**a**^ **(66.7%)**	**32**^**b**^ **(38.1%)**	0.001*
	**Negative**	**18**^**a**^ **(33.3%)**	**52**^**b**^ **(61.9%)**	
**PR status**	**Positive**	**40**^**a**^ **(70.2%)**	**35**^**b**^ **(42.2%)**	0.001*
	**Negative**	**17**^**a**^ **(29.8%)**	**48**^**b**^ **(57.8%)**	
HER2 status	Positive	7a (12.3%)	13a (15.5%	0.594
	Negative	50a (87.7%)	71a (84.5%)	
Treatment	Hormonal treatment	**27**^**a**^ **(75.0%)**	**23**^**b**^ **(47.9%)**	0.012*
	Chemotherapy	**7**^**a**^ **(19.4%)**	**25**^**b**^ **(55.6%)**	0.001*
	Anti HER2 therapy	2a (5.7%)	8a 17.4%)	0.114

*Statistically significant ^a,b^ Each superscript letter denotes a subtype whose column proportions do not differ significantly from each other at the 0.05 level

hpf – High power field (× 40 objective, field diameter 0.05 mm)

mpf – Medium power field (× 10 objective, field diameter 0.2 mm)

Percentages have been calculated to represent the prevalence of each clinical feature within each category of AR status

### Association of AR expression with IHC molecular subtypes

AR was expressed across all IHC molecular subtypes, with 50.6% of luminal, 29.4% of HER2 and 21.6% of TNBC subtypes showing AR-positivity. The association of AR status and the IHC molecular subtype is shown in Table 5. AR status showed a statistically significant association with the tumour IHC molecular subtype (*p* = 0.016) and AR-positivity was associated with the luminal subtype. Basal triple negative breast carcinomas showed an association with AR negativity.

**Table 5:** IHC Molecular subtype and AR expression

IHC molecular subtype	AR expression		***p***-value
	**Positive**	**Negative**	*p* = 0.016
**Luminal**	**44**^**a**^ **(77.2%)**	**43**^**b**^ **(51.2%)**	
HER2	5^a^ (8.8%)	12^a^ (14.3%)	
**Non-basal TNBC**	5^a^ (8.8%)	14^a^ (16.7%)	
**Basal TNBC**	**3**^**a**^ **(5.3%)**	**15**^**b**^ **(17.9%)**	

^a,b^Each superscript letter denotes a subtype whose column proportions do not differ significantly from each other at the 0.05 level

### Survival data of study sample

The mean duration of follow up was 46.70 (95% CI 46.495–46.905) months (3.89 years). One hundred and seven patients were alive and free of disease. There were 34 cases of early relapse. Twenty five patients died during this period. Of the nine that were living with disease, six had developed metastases and three had local recurrences.

Positive margin involvement was seen in ten cases (7.1%), of which eight were mastectomies and two were wide local excisions in which no subsequent mastectomy was performed. Three of the patients who died, had positive margin involvement at the time of primary surgery. All three had undergone mastectomy. The remaining seven patients were disease free at follow up.

#### AR status and survival

Androgen receptor status showed no significant association with OS (*p* = 0.351) and DFS (*p* = 0.840). Post-hoc calculations of the power of the study showed that the power of the study to detect a difference in DFS and OS between the two groups (AR+ vs AR-) with an alpha value of 0.05 was 4.6 and 11.2% respectively.

#### AR status and survival in sub groups

##### Menopausal status and survival

**Fig. 2:**
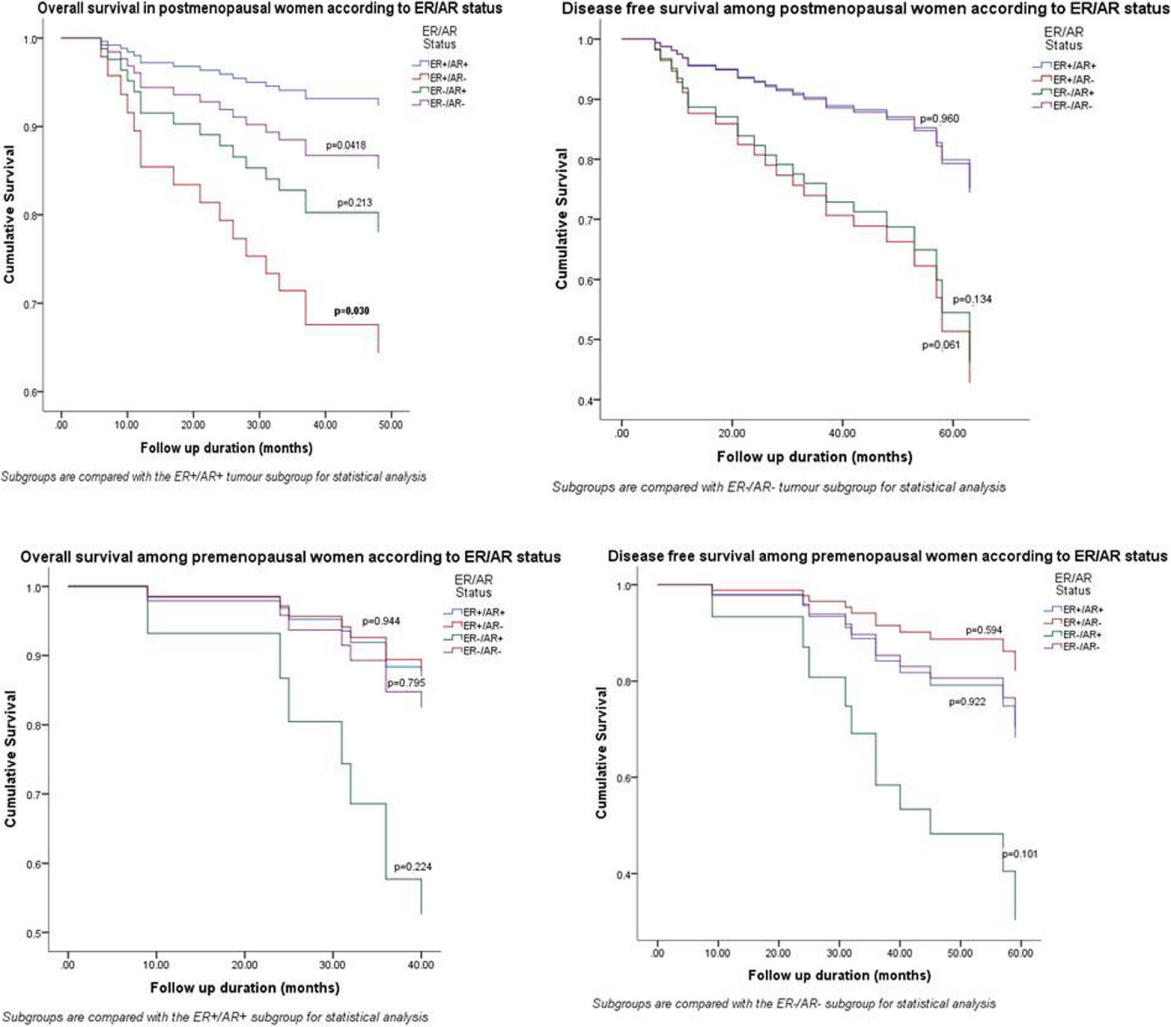
Impact of menopausal state and ER/AR status on overall and disease free survival

The tumours were evaluated in sub-groups based on the menopausal and ER/AR status. Postmenopausal women with ER-positive, AR-positive (ER+/AR+) tumours showed significantly better OS than postmenopausal women with ER-positive, AR-negative (ER+/AR-) tumours (*p* = 0.030) (Fig. 2). DFS did not differ among the different ER/AR expression subgroups of postmenopausal women. There was no statistically significant difference in OS or DFS among the different ER/AR expression subgroups among premenopausal women.

##### Histological grade and survival

**Fig. 3:**
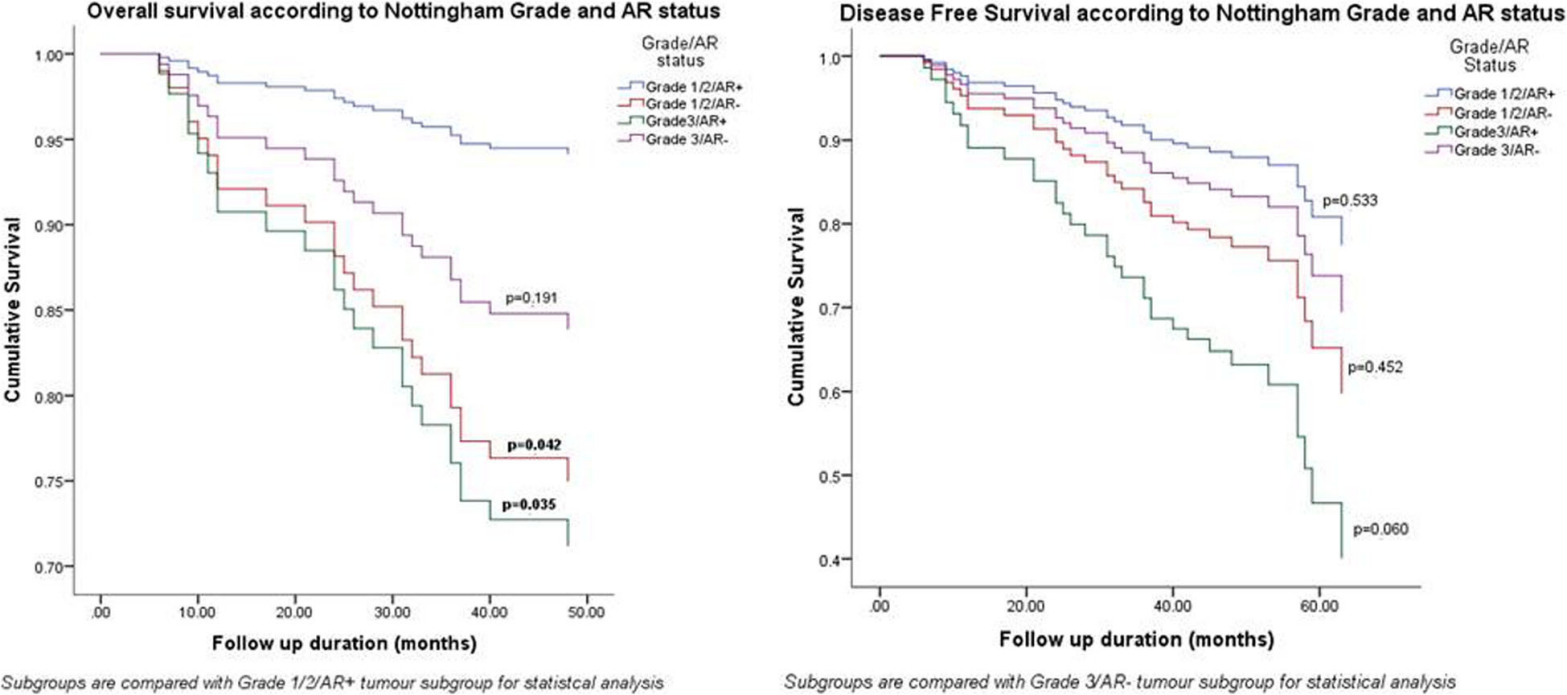
Impact of tumour grade and AR status on overall disease free survival according to tumour grade

Grade 3/AR-positive tumours had the worst OS and DFS. The OS (*p* = 0.035) and DFS (*p* = 0.022) of this subgroup was significantly different from the grade 1/2/AR-positive tumours. Additionally among grade 1/2 tumours, AR-positive tumours showed a significantly better OS (*p* = 0.042) than AR-negative tumours (Fig. 3).

##### ER/AR status and survival

**Fig. 4:**
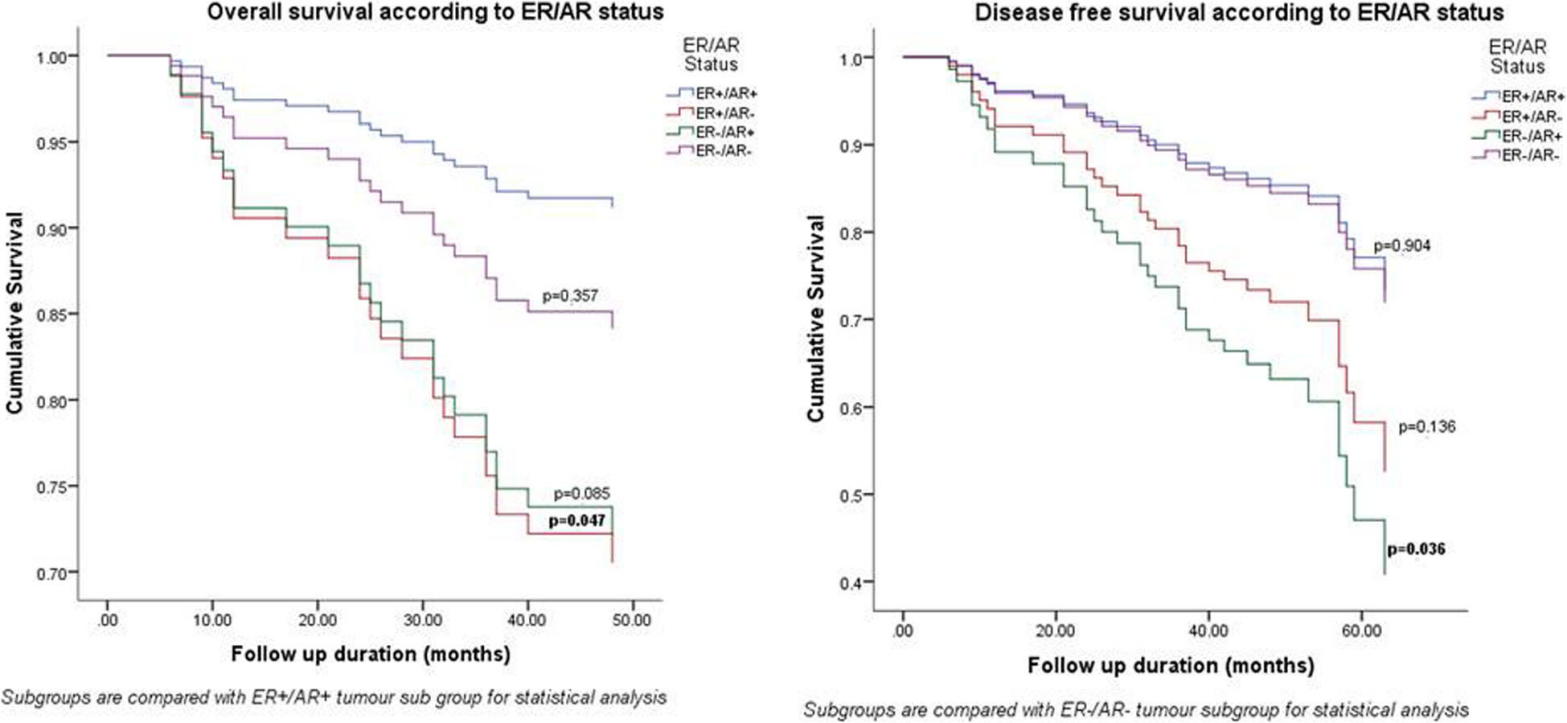
Impact of ER/AR status on overall and disease free survival

On univariate Cox regression analysis ER+/AR+ tumours showed significantly better OS than ER+/AR- tumours (*p* = 0.047) and ER-negative, AR-negative (ER−/AR-) tumours showed significantly better DFS than ER-negative, AR-positive (ER−/AR+) tumours (*p* = 0.036) (Fig. 4).

##### Molecular subtype and survival

The AR status of the tumour had no impact on either overall (*p* = 0.293) or disease-free survival (*p* = 0.826) when the tumours were categorized by molecular subgroup.

##### Multivariate analysis

On multivariate Cox regression the ER/AR status and TNM stage were the only factors predictive of OS and DFS. ER+/AR- tumours had a significantly worse OS than ER+/AR+ tumours (*p* = 0.015) and ER−/AR+ tumours had significantly worse DFS than ER−/AR- tumours (*p* = 0.014) (Tables 6 and 7).

**Table 6:** Multivariate Cox regression analysis of factors affecting overall survival

Prognostic factor	*p*-value	Hazard ratio	95% Confidence intervals
Age	0.115	1.036	0.991–1.083
TNM Stage	< 0.001		
**Stage I vs Stage III**	0.011	**0.062**	**0.007–0.534**
**Stage II vs Stage III**	< 0.001	**0.154**	**0.058–0.409**
Nottingham grade	0.360		
Grade 2 vs Grade 1	0.164	0.447	0.144–1.388
Grade 3 vs Grade 1	0.637	0.766	0.253–2.319
Margin involvement (Involved vs Uninvolved)	0.578	1.430	0.405–5.052
Molecular subtype	0.396		
HER 2 vs Luminal	0.456	0.505	0.084–3.042
TNBC vs Luminal	0.509	1.624	0.386–6.833
**ER/AR Status**	0.051		
**ER+/AR- vs ER+/AR+**	**0.015**	**5.500**	**1.395–21.685**
ER−/AR+ vs ER+/AR+	0.050	5.049	1.001–25.457
ER−/AR- vs ER+/AR+	0.345	2.256	0.417–12.207

**Table 7:** Multivariate Cox regression analysis of factors affecting disease free survival

Prognostic factor	*p*-value	Hazard ratio	95% Confidence intervals
Age	0.466	1.013	0.979–1.048
TNM Stage	< 0.001		
**Stage I vs Stage III**	0.007	**0.114**	**0.024–0.550**
**Stage II vs Stage III**	< 0.001	**0.181**	**0.077–0.427**
Nottingham grade	0.358		
Grade 2 vs Grade 1	0.193	0.518	0.193–1.394
Grade 3 vs Grade 1	0.773	0.862	0.313–2.371
Margin involvement (Involved vs Uninvolved)	0.970	0.976	0.281–3.385
Molecular subtype	0.283		
HER 2 vs Luminal	0.700	0.732	0.149–3.589
TNBC vs Luminal	0.282	2.090	0.545–8.012
**ER/AR Status**	**0.023**		
ER+/AR+ vs ER−/AR-	0.792	1.214	0.288–5.107
ER+/AR- vs ER−/AR-	0.079	3.308	0.870–12.580
**ER−/AR+ vs ER−/AR-**	**0.014**	**3.537**	**1.286–9.730**

## Discussion

The prevalence of AR expression has shown a wide variation, ranging from 37.04 to 77% in different studies [14–16, 26–28]. The prevalence of AR positivity was 40.8% among this cohort of Sri Lankan women with early breast cancer. The expression of AR is low in comparison to most studies. However studies conducted in Poland (43%) [26], Egypt (37.04%) [27] and India (56%) [15] have also reported low levels of AR expression. This wide variation in AR expression could be due to differences in tumour biology. Additionally studies have used different methods to determine AR expression (immunohistochemistry and gene expression profiling) and different cut offs (e.g. staining of ≥1% of cells or ≥ 10% of cells) to identify AR positive tumours by immunohistochemistry [10, 12, 29] Other factors that may have contributed to the low level of AR expression in this study include the exclusion of women who had received neo-adjuvant chemotherapy and false negativity due to the instability of the AR protein in stored tissue blocks.

Although some studies have shown that AR immune reactivity was related significantly to patient’s age and post-menopausal state [27], none of the clinical features that we studied showed an association with AR status. Pathological features associated with AR negativity included tumour necrosis involving > 50% of the tumor and a prominent lymphoid infiltrate at the tumour margin. These are features seen in basal like breast carcinoma [30] which was also shown to be associated with an AR – negative status.

Expression of AR receptor has been reported to vary between different IHC molecular subtypes. Studies performed in western settings such as in Germany, United States of America and Spain have reported an AR-positivity of 84–95% in ER-positive, 50–63% in ER-negative and 10–53% in TNBC [31]. The overall expression of AR was less across all tumour subgroups in the study population. However the same trend was demonstrated in the study and AR-positivity was present in 50.6% of luminal, 29.4% of HER2 and 21.6% of TNBC subtypes.

All patients were treated with surgery followed by adjuvant therapy, which included hormonal therapy, chemotherapy and radiation therapy, determined by standard treatment protocols. As expected given the association of AR positivity with ER positivity, AR positivity was associated with hormonal treatment and AR negativity with treatment with chemotherapy.

AR expression was not significantly related to the overall and disease-free survival in this study (mean duration of follow up - 46.70 months). However, many studies including a metanalysis of 22 studies involving 10,004 women [12] found that AR positivity was associated with favorable OS and DFS. The small sample size of this study may be a contributing factor for this difference.

The prognostic effect of AR expression may vary according to the age, menopausal status, and hormonal status of the tumour. Although the mean age was 55.46 years and most participants were in the post-menopausal age group, their age ranged widely from 29 to 77 years. The cases included both ER positive and ER negative cancers. This clinical heterogeneity needs to be taken into consideration, when interpreting the results of the group as a whole. Subsequent subgroup analysis was done according to menopausal status, tumour grade and ER status.

Postmenopausal women with ER+ tumours comprised a subgroup with limited clinical heterogeneity. We found that AR-positivity was associated with a better OS among postmenopausal women with ER-positive tumors. This same association was not seen among premenopausal women with ER-positive tumours. Androgen is thought to act as an anti-oestrogen in premenopausal women, but as an oestrogen agonist in postmenopausal women. Therefore it is possible that the prognostic implications of AR status may be dependent on the menopausal status. However the smaller numbers of women in the premenopausal group (44 premenopausal women vs 98 postmenopausal women) is a limitation that must be considered when interpreting the findings. Hu et al. who studied postmenopausal women also found AR expression to be associated with a more favorable prognosis in ER-positive tumors [10]. However the more recent breast international group trial 1–98 found that AR expression was not associated with prognosis in postmenopausal women with ER positive early breast cancer [32].

Among grade1/2 tumours, AR positive tumours showed a better prognosis than AR negative tumours. However this may be due to the fact that low grade tumours were more likely to be ER positive. ER+/AR+ tumours showed better OS than ER+/AR- tumours and ER−/AR+ tumours showed worse DFS than ER−/AR- tumours on both univariate and multivariate analysis. Other studies too have found that breast cancers with discordant ER/AR expressions (ER+/AR- or ER−/AR+) demonstrated a worse prognosis in comparison to breast cancers with concordant expression (ER + AR+ or ER−/AR -) in multivariable models [33]. As discussed above AR signaling antagonizes the growth stimulatory effects of ER signaling pathways in ER-positive cancers [6]. This may explain why women with ER-positive, AR-positive breast cancers have shown a better prognosis compared to all other AR/ER combinations [33]). It also highlights the importance of stratifying breast cancers according to the ER status when analysing the prognostic value of AR [33].

The implications of AR expression in HER2 amplified BCa and TNBC is uncertain. Some studies show no effect with AR-positivity [10, 34], some show poorer outcomes [12, 35] and others a good prognosis [7, 12]. AR expression was not associated with prognosis in the HER2 and TNBC IHC molecular subgroups of tumours in this study. However this may be due to the small numbers in these subgroups.

None of the patients studied had been treated with AR agonists or antagonists. Therefore the predictive value of AR status on treatment was not studied. The small sample size in the current study is a limitation. The power of the study is low due to the small sample size. The effects being investigated are also small. These factors increase the likelihood of both false negative and false positive results. This has to be taken into consideration in the interpretation of the results. Additionally the effect of adjuvant therapy also needs to be considered when assessing the impact on prognosis.

However despite these limitations, the finding that AR positivity was associated with a better prognosis in ER positive tumours, especially in postmenopausal women is in keeping with other studies. AR positivity was associated with poorer outcomes in ER negative tumours. These findings are supportive of the use of androgen agonists in ER+/AR+ tumours and AR antagonists in ER−/AR+ tumours [6, 11]. Large studies have provided conflicting evidence on the prognostic impact of AR status in early breast carcinoma, particularly in post-menopausal women [10, 32]. This is the first study on AR expression in breast carcinoma in Sri Lanka and despite the limitations, these findings add to the evidence that AR positivity is a widespread aspect of breast cancer that deserves therapeutic attention and further study.

## Conclusions

The histological and IHC molecular tumour characteristics of this BCa patient cohort from Sri Lanka, were mostly similar to the findings from other regions of the world including Europe and United States of America [26, 28, 36, 37]. The prevalence of AR positivity was lower than most studies in Europe and America which may be a result of regional differences in tumour biology. AR positivity was associated with ER and PR positivity and the IHC luminal subtype. The prognostic effect of AR was dependent on ER expression, with concordant ER/AR expression being predictive of better OS and DFS. AR positivity was associated with a better prognosis among women with ER-positive tumors, especially in the postmenopausal age group.

Currently, where the androgen receptor (AR) is emerging as a new biomarker and a potential new therapeutic target in the treatment of BCa [11], the study findings may have prognostic and therapeutic implications for treatment protocols including androgen receptor agonists or antagonists in appropriate clinical settings.

## Data Availability

The datasets generated and analyzed during the current study are not publicly available since explicit consent for data publication was not obtained from participants at the time of the study and multiple indirect identifiers are included in the data base. However it could be made available from the corresponding author on reasonable request.

## Abbreviations

AR: Androgen Receptor
BCa: Breast Carcinoma
CI: Confidence Interval
DCIS: Ductal Carcinoma in situ
DFS: Disease Free Survival
ER: Oestrogen Receptor
hpf: High power field
IHC: Immunohistochemical
mpf: Medium power field
OS: Overall Survival
PR: Progesterone Receptor
TNBC: Triple negative breast carcinoma
